# The Effects of Body Mass on Dung Removal Efficiency in Dung Beetles

**DOI:** 10.1371/journal.pone.0107699

**Published:** 2014-09-17

**Authors:** Beatrice Nervo, Claudia Tocco, Enrico Caprio, Claudia Palestrini, Antonio Rolando

**Affiliations:** Department of Life Sciences and Systems Biology, University of Torino, Torino, Italy; Landcare Research, New Zealand

## Abstract

Understanding of the role of body mass in structural-functional relationships is pressing, particularly because species losses often occur non-randomly with respect to body size. Our study examined the effects of dung beetle body mass on dung removal at two levels. First, we used the lab experiment to evaluate the efficiency of eight dung beetle species belonging to two functional groups (tunnelers, dwellers) on dung removal. Second, the same species employed in the lab were used in field mesocosms to examine the effects of the two functional groups on dung removal maintaining realistic differences in the total body mass between tunneler and dweller assemblages. Furthermore, the experimental assemblages contained one and four species within each functional group, so the effect of body mass heterogeneity was examined. We used a statistical approach (offset method) which took into account *a priori* constraints due to the study design allowing us to analyse the effect of larger species in mesocosm style experiments. Body size played a crucial role in dung removal: large beetles were more efficient than small ones and the percentage of removed dung increased with higher body mass heterogeneity. Tunnelers were more efficient than dwellers over both short and long time periods (one month and one year). Significant effects of dwellers were found only after one year. Moreover, our study showed that not including the body mass as an offset in the model resulted in sometimes different results, as the offset expresses dung removal independently of the body mass. This approach confirmed that body size is likely a pivotal factor controlling dung removal efficiency at multiple levels, from single species to overall dung beetle assemblages. Even though other specific traits should be examined, this study has begun to address the consequences of losing individuals with specific traits that are especially sensitive to perturbations.

## Introduction

The last two decades of intensive research have provided compelling evidence for a link between biodiversity and ecosystem functioning (BD-EF). In particular, BD-EF research has concentrated on species richness and on the impact of anthropogenic environmental changes on ecosystem functions and services [1], [2], [3]. Species richness, one of the key components of biological diversity, may indeed be important for maintaining functional processes due to interspecific differences in how species process resources, affect the physical environment, and interact with one another [4]. However, the perceived artificiality of the random community assemblages used to create various experimental gradients of diversity is a constant criticism of experimental BD-EF research [5]. Several authors have emphasized that natural and anthropogenic diversity gradients show non-random patterns in the order and characteristics of species lost [6], [7], [8], [9]. Such extinction bias raises questions about how useful inferences from random-assembly designs will be for informing conservation efforts. Since species and their associated functions are currently being lost at an unprecedented rate, understanding of the role of body mass in structural-functional relationships is pressing, particularly because species losses occur non-randomly with respect to body size [10]. Interspecific differences in body mass can have potentially profound effects across multiple scales of biological organization, since many life-history traits are body mass correlated. Body mass indeed may represent an indicator of the niche of each species and by extension of the entire ecological network [10]. For this reason, in BD-EF studies on bacteria, protists and animals, we need to take into account the body mass of the individuals within the biodiversity entity (e.g. species or functional groups) because ecosystem processes may depend not only on ecological traits but also on the metabolic rate of the individuals [3]. According to this scenario, there may be different patterns in the consumption of resources within the same functional group dependent on whether is composed of small or large species, since the metabolic rate per unit body mass is thought to decrease with increasing body size [11], [12].

Dung beetles (Coleoptera: Scarabaeoidea) play a key role in dung removal and soil processes, and as such they are considered important in terms of providing ecosystem functions and services. Largely coprophagous, they use three broad nesting strategies driving a series of ecological processes such as dung removal, nutrient cycling, bioturbation, plant growth enhancement, secondary seed dispersal, trophic regulation, parasite suppression and fly control [13]. Dweller species lay eggs and brood their larvae inside the dung mass itself, or just at the soil-dung interface. Tunneler species bury brood balls in vertical chambers in close proximity to the original deposition site. Roller species transport dung balls some distance away, before burial beneath the soil surface. These varied patterns of consumption and relocation of dung are included within functional diversity, another relevant component of biological diversity characterizing species assemblages. Here, we focused on dung beetle assemblages of alpine pastures, where tunnelers are represented by several medium-large species, while dwellers are usually much smaller. There is great concern about the decline of dung beetles in Western Europe [14], [15], especially with regard to large body sized species. For example, in Finland there has been a significant loss of large, tunneling Geotrupidae beetles (one out of three species now regionally extinct) and tunnelling *Onthophagus* (two out of three species lost) [16], in the Mediterranean region with a decline of large roller species populations [17], and the Padana Plain (northern Italy) has experienced the virtual extinction of at least one species of *Scarabaeus* and three species of *Gymnopleurus* [18]. Moreover, some studies have shown the ecological role of dung beetle assemblages in Finnish and UK pastures [16], [19], [20], but there is limited research on the ecological role of dung beetles in the Italian Alps, where land-use changes are affecting biodiversity and threatening local ecosystem services [21].

Our study examines the effects of dung beetle body mass on dung removal at two levels (individual species and the functional group). First, we used the lab experiment to evaluate the efficiency of eight dung beetle species belonging to two functional groups (tunnelers and dwellers) on dung removal. Second, the same species employed in the lab were used in field mesocosms to examine the effect of the two functional groups on dung removal. Since the experimental assemblages contained one and four species within each functional group, the influence of body mass heterogeneity on dung removal was analysed too. Finally, we developed an original statistical approach which took into account *a priori* constraints due to the study design allowing us to analyse the effect of larger species in mesocosm style experiments and compare assemblages with different body mass.

## Material and Methods

### Study area and species collection

The study area was a pasture dominated by graminaceous plants, which is located in a private protected area (Oasi Zegna), included in the Sessera Valley (45°40′16″N; 8°05′07″E, N-W Italy, 1400 m a.s.l.). Oasi Zegna (www.oasizegna.com), gave permission to conduct this research and collect dung beetles. No endangered or protected species were involved in the field studies. The climate is temperate sub-alpine with a mean annual temperature of 10°C and a mean annual rainfall of 1800 mm. A preliminary study in this area showed that the dung beetle assemblage is characterized by 27 species belonging to two families and to two functional groups (Scarabaeidae and Geotrupidae as tunnelers and Aphodiinae, a subfamily of Scarabaeidae, as dwellers) [22]. Field and lab experiments were carried out in June 2012 and 2013. In both years, four tunneler species (*Anoplotrupes stercorosus* (Scriba, 1796), *Trypocopris pyrenaeus* (Charpentier, 1825), *Onthophagus fracticornis* (Preyssler, 1790), *Geotrupes stercorarius* (Linnaeus, 1768)) and four dweller species (*Teuchestes fossor* (Linnaeus, 1758), *Acrossus depressus* (Kugelann, 1792), *Parammoecius corvinus* (Erichson 1848), *Acrossus rufipes* (Linnaeus, 1758)) were collected by hand or using dung-baited pitfall traps. Collected beetles were transferred to terraria until the start of the experiments. A minimum of ten dried individuals of each species were weighed to estimate body mass using an analytical balance (0.01 mg). Measured body mass was used as a proxy for body size. Fresh cattle dung was collected for the experiments from a closed barn in a livestock farm and was mixed to make it of uniform consistency; no insects nor larva were found in the dung samples. The cattle-grazed pasture had not been treated with anthelmintics such as ivermectin.

### Lab experiments

Lab experiments were carried out to test the efficiency of dung removal for each of the eight species. Two individuals of each species were added to a 25 cm diameter terrarium with 45 g dung for dwellers and 90 g dung for tunnelers (5×5 cm dung size). When possible, beetles were sexed to ensure the presence of a male and a female in the pair. The amount of dung provided to dwellers was halved compared to tunnelers because of their smaller size. There were eight replicates for each species (four with *A. rufipes* because too few individuals were collected), and four controls only with dung. Each terrarium was covered by nylon mesh cloth to prevent beetles from escaping and others from entering. The residual dung was collected, oven-dried and weighed after 80 hours from the beginning of the experiment. This interval was set because it was the time taken by the most efficient species in monoculture (*A. stercorosus*) to remove most of the dung (Pers. Obs). This experimental protocol allowed the evaluation of the dung removal rate by adults only, because no larvae could hatch from eggs in such a short time.

### Field experiments

The same eight dung beetle species were used to set the experimental field treatments in which we compared assemblages with one and four species of tunnelers (T1, T4) and dwellers (D1, D4) in order to evaluate the role of the functional groups and body mass heterogeneity in dung removal. Controls (C) were set up by using dung without beetles. The number of individuals was varied in each treatment in order to maintain the same total body mass within the tunneler treatments, and within the dweller treatments. The species used for the monocolture (10 individuals of *A. stercorosous* for tunneler treatments- T1, and 24 individuals of *T. fossor* for dweller treatments- D1) were chosen based on two criteria: (i) the abundance of the species in the study area; (ii) the dung removal efficiency showed in the lab experiment. In tunnelers, *A. stercorosus* was the most efficient and the most abundant species; in dwellers, *A.rufipes* showed the highest efficiency in dung removal, but unfortunately it was the less abundant. For this reason we decided to use *T. fossor* in monoculture which was the second most efficient species in terms of dung removal. The total body mass of tunnelers in the treatments (1.634 g) was doubled compared to dwellers (0.864 g) which reflected approximately the proportion of individuals between the two functional groups under natural field conditions in the Alps [22] (Table 1). We also took into account body mass heterogeneity, BH, calculated as σ/μ, where σ is the standard deviation and μ is the mean of the total body mass in each treatment. Maintaining differences in body mass between tunnelers and dwellers, instead of holding total dung beetle body mass constant (as per [19]), and using small and large species in our mesocosms allowed a more realistic representation of the natural composition of dung beetle assemblages in the study area. Fifty frames (PVC pipes, 40 cm diameter, 15 cm height) were fixed in the ground to a depth of 10 cm and randomly assigned to the experimental treatments and control. Two experimental units were considered: twenty-five frames were used to analyze dung removal efficiency after one month and twenty-five to analyze it after one year. Five replicates were considered in each experimental unit (4 treatments and 1 control×5 = 25). The control has served to detect for the presence of earthworms or other dung consumers. In according to other studies such as [19], [23], 600 g of dung and the dung beetles were added to the treatments and each pipe was covered by nylon mesh cloth. Dung residuals were collected, oven-dried, and weighed respectively after one month and one year in each experimental plot.

**Table 1:** Dung beetle species used in the experiments.

Functional groups and species	Total number of beetles	Mean body mass (mg)	N of individuals for each frame			
			T1	T4	D1	D4
**Tunnelers**						
*Anoplotrupes stercorosus*	140	163.4	10	4		
*Geotrupes stercorarius*	20	220.31		2		
*Trypocopris pyrenaeus*	20	165.13		2		
*Onthophagus fracticornis*	140	15.54		14		
**Dwellers**						
*Teuchestes fossor*	420	36			24	18
*Acrossus depressus*	60	7.8				6
*Acrossus rufipes*	20	34.19				2
*Parammoecius corvinus*	400	2.06				40
**Body mass heterogeneity (BH)**			18.3	111.1	14.9	118.2

Total number of individuals used in the field experiment, mean dry body mass, body mass heterogeneity and number of individuals used in each treatment frame per species (10 replicates for each frame, five for short-term effects and five for long-term effects). Key to treatment codes: T1 = one tunneler species; T4 = four tunneler species; D1 = one dweller species; D4 = four species of dwellers.

### Statistical analysis

The effects of each tunneler and dweller species on the proportion of dung removed in lab experiments were tested using generalized linear models (GLM). Grams of residual dung were expressed as a percentage of removed dung as follows: [1-(grams of residual dry dung/grams of starting dry dung)]*100. Species was modeled as an independent categorical variable. The same analysis was used to assess the effects of different functional groups and body mass heterogeneity on the proportion of dung removed in the field. The experimental treatments (and the control) and body mass heterogeneity within the treatments were modeled as independent categorical variables (low and high body mass heterogeneity). Body mass heterogeneity, expressed as the coefficient of variation of the body mass within each treatment, was tested as a numerical variable too (BH). The body mass of each species used in the lab experiments was added to the models as an offset in order to statistically adjust for body size, as there was a significant correlation between dung removal rates and body mass (Table S1 in Appendix S1). Body mass *per se* is likely to be a key determinant of dung removal in such systems (e.g. [16], [24]), hence it was statistically adjusted for in order to better compare the actual effects of different species on dung removal. In the field experiments, differences in the total body mass between tunneler and dweller treatments were therefore considered using the total body mass within the treatments as an offset. The offset term adjusts the model's dependent variable taking into account possible bias linked to the data collection or sample design [25], [26]. Visual inspection of frequency distributions and Shapiro-Wilk tests confirmed the normality of errors in most cases. The gamma distribution was specified otherwise. *Post-hoc* Interaction Analysis calculated pairwise differences between the species (lab experiment) and the treatments (field experiment) using the Chi-square test (Package Phia in R [27]) and correcting the p-values according to the Holm method. All analyses were carried out using R 3.0.3. [28].

## Results

### Lab experiments

**Figure 1:**
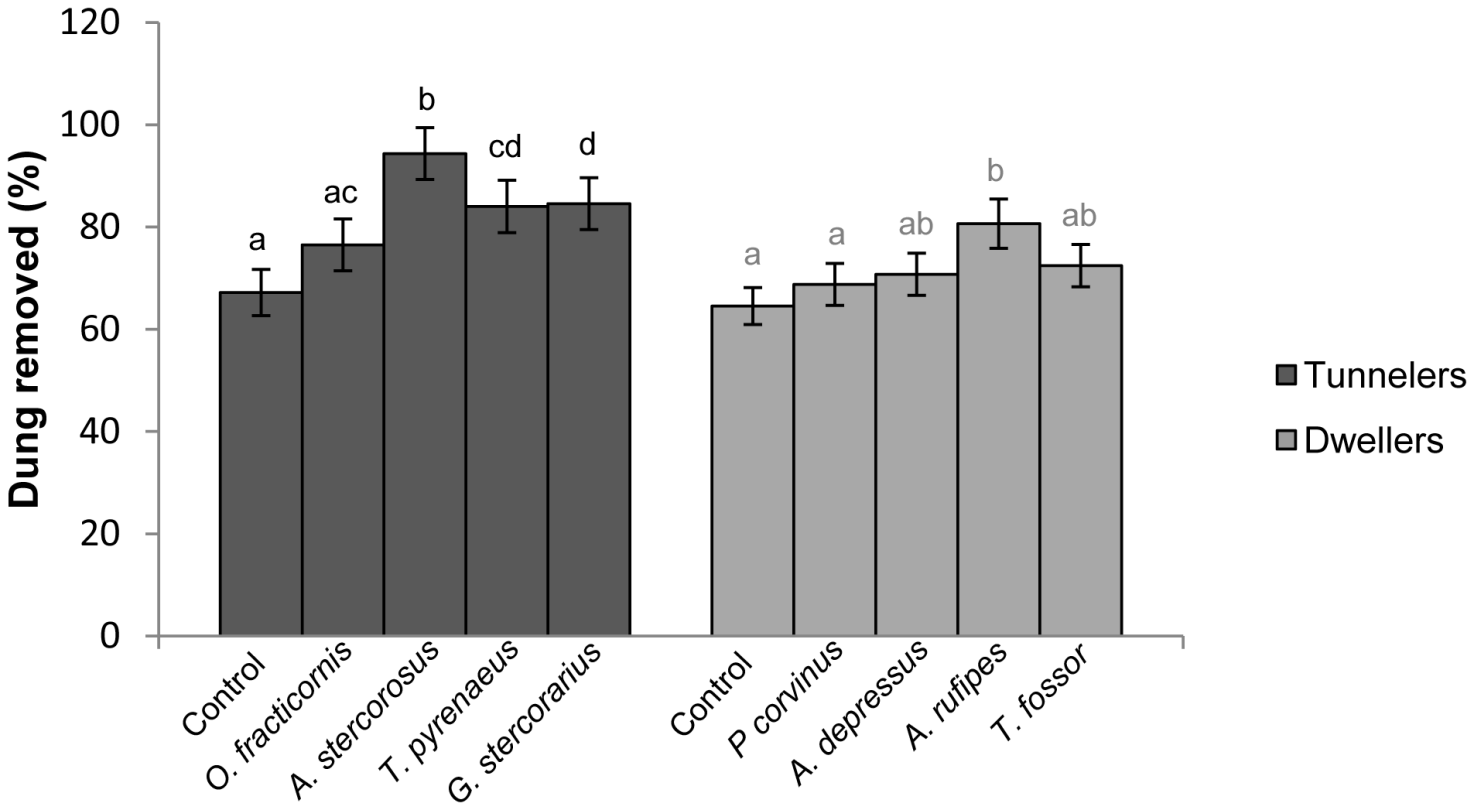
Lab experiment. Parameter estimates of dry dung removed (%) after 80 hours in tunneler (left) and dweller (right) species, derived from GLMs. Species are ordered in terms of their body mass (ascending order). Letters over the error bars indicate the differences among species and control.

Dung removal efficiency increased with increasing body mass both in tunnelers (p<0.01) and dwellers (p<0.01) (Table S1 in Appendix S1). Larger species removed more dung per unit of weight, i.e. they were more efficient even when accounting for body size. Within tunneler species, *A. stercorosus* removed a significantly higher proportion of dung compared to the other three tunneler species when adjusting for body size (Table 2, Figure 1). All the tunneler species removed a significant part of the dung mass compared to controls (removed dung varied between 5% and 33%), with the exception of *O. fracticornis* (9%–10%). Post-Hoc Interaction tests showed that *A. stercorosus* was the most efficient species compared to the other three tunnelers and that *G. stercorarius* removed more dung than *O. fracticornis*. *A. rufipes* showed a high efficiency in dung removal (12–30%), while none of the other dweller species removed a significant part of the dung mass compared to controls after 80 hours (0–7%) (Table 2, Figure 1). The Interaction test between dweller species was significant only between *A. rufipes* and *P. corvinus* (Table 2).

**Table 2:** Effects of tunneler and dweller species on dung removal in the lab.

TUNNELER SPECIES				DWELLER SPECIES			
Reference level: Control				Reference level: Control			
Distribution: Gaussian	Estimate	t value	*p*	Distribution: Gamma	Estimate	t value	*p*
Intercept	67.2	14.83	***	Intercept	64.55	17.82	***
*Anoplotrupes stercorosus*	27.16	5.36	***	*Parammoecius corvinus*	4.23	1.03	NS
*Geotrupes stercorarius*	17.38	3.43	**	*Acrossus depressus*	6.21	1.50	NS
*Onthophagus fracticornis*	9.31	1.84	NS	*Teuchestes fossor*	7.9	1.90	NS
*Trypocopris pyreneaus*	16.83	3.28	**	*Acrossus rufipes*	16.1	3.33	**

*Interaction Test*	Value	Chisq	*p*	*Interaction Test*	Value	Chisq	*p*
*A. stercorosus - G. stercorarius*	9.78	9.32	*	*A. rufipes - P. corvinus*	11.87	10.08	*
*A. stercorosus - O. fracticornis*	17.85	31.04	***	*A. rufipes - A. depressus*	9.89	6.89	NS
*A. stercorosus - T. pyrenaeus*	10.33	9.69	*	*A. rufipes - T. fossor*	8.19	4.67	NS
*G. stercorarius - O. fracticornis*	8.07	6.34	*	*P. corvinus - A. depressus*	−1.98	0.51	NS
*G. stercorarius - T. pyrenaeus*	0.54	0.03	NS	*P. corvinus - T. fossor*	−3.67	1.71	NS
*O. fracticornis - T. pyrenaeus*	−7.52	5.15	NS	*A. depressus - T. fossor*	−1.69	0.35	NS

Factor estimates and statistical significance (GLM) for the dung removed in relation to the tunneler (sample size = 33) and dweller (sample size = 30) species. This table shows the results of the statistical models with the “body mass” of each species as an offset. Significance codes:

‘***’p<0.001;

‘**’ p<0.01.

‘*’ p<0.05.

### Field experiments

**Figure 2:**
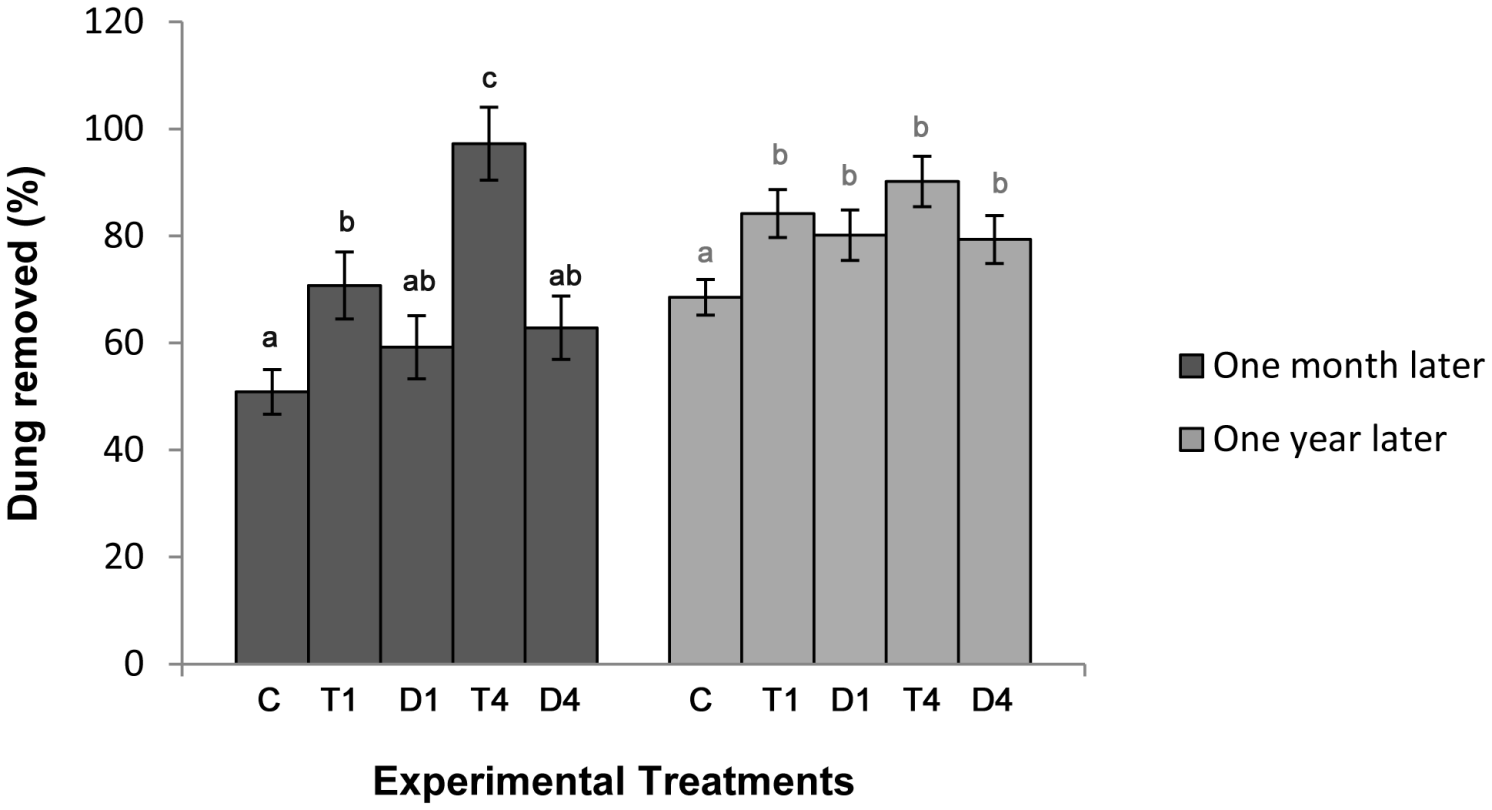
Field experiment. Parameter estimates of dry dung removed (%) after one month and one year in the experimental field treatments, derived from GLMs. Letters over the error bars indicate the differences among treatments and control.

The percentage of dung removed in tunneler treatments compared to control was significantly higher after one month and in the treatments of both functional groups after one year (Figure 2). The Interaction test between treatments showed that T4 removed significantly more dung than T1, D1 and D4 after one month, while differences were no more significant after one year. Comparisons between dung removal efficiency of the two functional groups showed that tunnelers removed a significantly higher proportion of dung both after one month and one year (Table 3). Tunneler treatments with high body mass heterogeneity (four species, T4) removed significantly more dung compared to those with low body mass heterogeneity (one species, T1) after one month, but these differences were no longer significant after one year. Even though percentage of removed dung tended to increase in dweller treatments with four species compared to controls after one month, differences between low and high body mass hetrogeneity (D1, D4) were not significant either one month or one year later. The percentage of dung removed increased significantly with higher body mass heterogeneity (BH) after one month but no longer after one year (Table S1 in Appendix S2). Both the models with offset (Tables 2–3) and without offset (Table S2 in Appendix S1 and Table S2 in Appendix S2) differed for the value of the estimates and levels of significance. The most evident difference regarded the effect of the treatment D4 after one month, which removed significantly higher proportion of dung in the models without offset (Table S2 in Appendix S2).

**Table 3:** Effects of dung beetles on dung removal after one month and after one year in the field experiment.

AFTER ONE MONTH				AFTER ONE YEAR			
FIELD TREATMENTS				FIELD TREATMENTS			
*Reference level: Control*				*Reference level: Control*			
*Distribution: Gaussian*	Estimate	t value	*p*	*Distribution: Gaussian*	Estimate	t value	*p*
Intercept	50.85	12.16	***	Intercept	68.54	20.56	***
T1	19.89	3.17	**	T1	15.63	3.49	**
D1	8.35	1.41	NS	D1	11.6	2.46	*
T4	46.39	6.79	***	T4	21.64	4.59	***
D4	11.99	2.03	NS	D4	10.79	2.41	*

*Interaction Test*	Value	Chisq	*p*	*Interaction Test*	Value	Chisq	*p*
T1-D1	11.54	3.38	NS	T1-D1	4.02	0.81	NS
T1-T4	−26.49	13.77	**	T1-T4	−6.00	1.8	NS
T1-D4	7.9	1.59	NS	T1-D4	4.84	1.32	NS
D1-T4	−38.08	31.04	***	D1-T4	−10.04	4.53	NS
D1-D4	−3.64	0.38	NS	D1-D4	0.81	0.03	NS
T4-D4	34.39	25.39	***	T4-D4	10.85	5.89	NS

FUNCTIONAL GROUP				FUNCTIONAL GROUP			
*Reference level: Dwellers*				*Reference level: Dwellers*			
*Distribution: Gaussian*	Estimate	t value	*p*	*Distribution: Gaussian*	Estimate	t value	*p*
Intercept	61.02	17.5	***	Intercept	79.69	33.42	***
Tunnelers	21.08	3.88	**	Tunnelers	7.15	2.12	*

BODY MASS HETEROGENEITY				BODY MASS HETEROGENEITY			
Tunnelers (T1 vs T4)				Tunnelers (T1 vs T4)			
*Reference level: T1*				*Reference level: T1*			
*Distribution: Gaussian*	Estimate	t value	*p*	*Distribution: Gaussian*	Estimate	t value	*p*
Intercept	70.73	20.36	***	Intercept	85.8	19.95	*
T4	26.49	4.99	**	T4	6.02	0.95	NS

BODY MASS HETEROGENEITY				BODY MASS HETEROGENEITY			
Dwellers (D1 vs D4)				Dwellers (D1 vs D4)			
*Reference level: D1*				*Reference level: D1*			
*Distribution: Gaussian*	Estimate	t value	*p*	*Distribution: Gaussian*	Estimate	t value	*p*
Intercept	60.06	20.43	***	Intercept	80.14	39.71	***
D4	3.62	0.87	NS	D4	−0.83	−0.3	NS

Treatment factor estimates and statistical significance (GLM) for the dung removed in relation to the experimental treatments (sample size = 22), functional groups (sample size = 18) and body mass heterogeneity (sample size = 9). This table shows the results of the statistical models with the “total body mass” within the treatments as an offset. Key to treatment codes: T1 = one tunneler species; T4 = four tunneler species; D1 = one dweller species; D4 = four species of dwellers.

Significance codes:

‘***’p<0.001;

‘**’ p<0.01.

‘*’ p<0.05.

## Discussion

### Functional group identity

Both our lab and field experiments clearly demonstrated that tunnelers were more efficient than dwellers in dung removal. Even though both groups in the field showed a significant removal effect after one year, dwellers appeared unable to provide a significant contribution to this ecosystem function compared to tunnelers in the short term, after one month. Dwellers take more time to remove dung due to their nesting behavior: larvae bred inside the dung consume the more fibrous material, while adults feed on the liquid component [13]. Only first and second instars were found in the dung collected after one month, meaning that they would have continued to consume dung, with an apparent effect over a longer period of time. Indeed, [29] showed that *A. rufipes* larvae are particularly active during the third instar when larvae are known to dig tunnels and display kleptoparasitic behavior [30]. As a result, the dung attains a much more loose and friable texture than material of a similar age which has not been worked through by larvae. For the same reason, with the notable exception of *A. rufipes*, no significant effects of dwellers were found in the lab experiments: an 80 hour interval was not long enough to highlight the delayed effects of dwellers. In contrast, tunnelers were more efficient in providing dung removal in the short term because adults not only feed on dung, but they also remove dung to provision nests in tunnels excavated in the ground beneath the dung pad. The long term effect of dung beetles on dung removal allows an evaluation of pasture quality after one year, before the beginning of the new season of pastoral activity. It is known that an area up to 12 times larger than the dung pad itself will remain ungrazed by livestock for several months, and even up to a year, if it remains in situ. This is due to its strong odour and to plant lignification [31]. However, if the dung pad is quickly buried or consumed, such areas are not avoided, hence there will be a larger area of pasture available for grazing [32].

### Body mass

This study confirmed that body size is likely a pivotal factor controlling dung removal efficiency at multiple levels, from single species to overall dung beetle assemblages. Our lab experiment, as elsewhere [23], [33], [34], [35], showed that body mass is an ecologically relevant characteristic because smaller beetles had relatively little effect on dung removal. For example, the effect of *O. fracticornis* on dung removal was certainly lower compared to the other tunnelers used in the experiment and it was not significant after 80 hours (i.e. its body mass was 1/14 than that of the largest *G. stercorarius*). Even though body size is considered an universally important trait influencing biodiversity-ecosystem function relationships within the detritivore trophic level [36], [37], we expect that other species-specific traits in addition to body mass may influence dung utilization when differences between species' size are relatively small. The lab experiment, indeed, showed that *A. stercorosus* was the most efficient species in dung removal even if it was 30% smaller than *G. stercorarius*. Body mass may also explain differences between functional groups because local tunnelers were usually much larger than dwellers (i.e. *G. stercorarius* was 106 times larger than *P. corvinus*). Field experiments confirmed that large beetles, represented mainly by tunnelers in our alpine study area, have a dominant effect on dung removal. The reduced efficiency in dung removal due to a smaller body size could be compensated by a higher density of individuals [38]. However, even if dwellers are the dominant component of the Sessera Valley, large tunnelers showed high abundances [22] and consequently higher total body mass compared to smaller dwellers in the pastures of our study area. This suggests that the loss of larger species, which are often more prone to extinction [16] may have important consequences on the ecosystem functioning. Furthermore, by enlarging field mesocosms to also include geotrupid species, our study differed to that of [19], who considered only relatively small *Aphodius* and *Onthophagus* species in order to maintain the total body mass constant. The inclusion in the field treatments of larger species such as *G. stercorarius* and *A. stercorosus*, which are among the most abundant species in the pastures of the Sessera Valley [22], allowed a more realistic representation of the composition of the dung beetle community in the study area. Our results, which showed an enhancement of dung removal with higher body mass heterogeneity (BH), indicated that this may be an important driver in promoting dung burial and removal. Focusing on the treatments with one and four species within the same functional group (low and high body mass heterogeneity), we found that *A. stercorosus*, the most efficient species in dung removal as demonstrated by our lab experiments, increased its efficiency in the field mesocosms when it was in association with the other three tunneler species (T4). Since the most efficient species (and not the less efficient) was used as a term of comparison, this result suggests that body mass heterogeneity and probably the number of species may have increased ecosystem functioning in the short-term. This result was confirmed also by the increase in the percentage of dung removed after one month in relation to higher body mass heterogeneity (BH). Body mass heterogeneity may promote resource partitioning and facilitation, leading to a more efficient acquisition of resources. The form of resource partitioning and facilitation that we suppose consists of different patterns of food selection for different species due to their different ecological traits (i.e. body size) [39] and to the mutual benefit from one another through biophysical interactions of the resource [40]. Moreover species with different body size will also have different metabolism and consumption rates of resources [11] which may promote a more efficient dung removal in heterogeneous assemblages.

However, differences between dweller treatments with one and four species were not significant both one month and one year later. The slight tendency in treatment D4 (four dweller species) to remove a greater percentage of dung compared to the control after one month may have been attributed to the presence of *A. rufipes* which was the only dweller able to remove a significant part of the dung mass in the lab experiments.

### Offset feature

Finally, using the offset term in the models allowed us to include the larger species in our study and compare treatments with different total body mass. The use of an offset allowed us to adjust the parameters of our models taking into account complicating effects of variations in body mass on the relationship between treatments (or species in the lab experiment) and dung removal. This approach represents one of the most novel aspect of our study because it presents a new way of considering the effects of larger species in mesocosm-style experiments. To our knowledge, the GLM offset feature has been over looked in other studies which compared assemblages with different total body mass. In our case, even though the results with offset (Tables 2–3) and without offset (Table S2 in Appendix S1, Table S2 in Appendix S2) were similar, the omission of total body mass from our models resulted in more notable differences in parameter estimates and significance levels of the field experiment than those of the lab experiment. In particular, the dweller treatment with high heterogeneity (D4) was significant compared to control when offset was omitted, but was not significant when taking into account variations in total body mass of the dung beetles among the treatments. This shows that results may differ depending on whether total dung removal or dung removal efficiency are being considered since different assemblages perform not only according to their mean specific traits, but also to the total body mass of the assemblage. In the lab experiment, models with or without body mass as an offset showed slight differences, highlighting that larger species removed more dung even when accounting for body size. Consequently, the performance of our species did not depend exclusively on the trait considered (i.e. body size), but probably also on the metabolic rates of the individuals and other species-specific traits which need to be investigated further.

As a caveat, we recognise the limitations of our study which considered a single study area in the Alps, and which was conducted on a limited number of species which were all tested in monoculture, but only in the lab. Accordingly, while we have not found differences in dung removal within dweller treatments under present local conditions, they may emerge under others, as shown by [19]. Admittedly, this design left many species combinations unstudied mainly for lack of sufficient number of individuals. Nonetheless, the trait-based approach used in our study could start to address the consequences of losing individuals with specific traits that are especially sensitive to particular perturbations. For instance, habitat loss usually affects large-bodied organisms disproportionately [3], [41]. Future studies should be aimed at considering other specific traits beyond body mass, and at analyzing multiple functions. In this framework, only appropriate management aimed at preserving heterogeneous dung beetle assemblages in body size (especially medium and large tunneler species) can warrant the maintenance of ecosystem services in the Alps, threatened by the land use changes driven mainly by the overall decline of traditional agro-pastoral systems [42], climate change [43], and human leisure activities [44], [45].

## Supporting Information

Appendix S1 **Details of GLMs used for the lab experiment without the offset term.** Factor estimates and statistical significance (GLM) for the dung removed in relation to the tunneler (sample size = 33) and dweller (sample size = 30) species. Also, models with body mass of each species in relation to the percentage of removed dung are presented. Significance codes: ‘***’p<0.001; ‘**’ p<0.01 ‘*’ p<0.05.
(XLSX)

Appendix S2 **Details of GLMs used for the field experiment without the offset term.** Factor estimates and statistical significance (GLM) for the percentage of removed dung in relation to the experimental treatments (sample size = 22) after one month and one year. Also, models with body mass heterogeneity in relation to the percentage of removed dung are presented. Significance codes: ‘***’p<0.001; ‘**’ p<0.01 ‘*’ p<0.05.
(XLSX)
